# 

**Published:** 1863-08

**Authors:** 


					﻿THE
DENTAL REGISTER
OF THE WEST.
EDITED BY
J. TAFT AND GEO. WATT.
AUGUST-1863.
MONTHLY:
Three Hollars per Annum, in advance.
CINCINNATI:
J. TAFT, PUBLISHER.
J. Taft, Dentist, 172 Race Street.
				

